# rBAN: retro-biosynthetic analysis of nonribosomal peptides

**DOI:** 10.1186/s13321-019-0335-x

**Published:** 2019-02-08

**Authors:** Emma Ricart, Valérie Leclère, Areski Flissi, Markus Mueller, Maude Pupin, Frédérique Lisacek

**Affiliations:** 1 Proteome Informatics Group, SIB Swiss Institute of Bioinformatics, CMU, Rue Michel-Servet 1, 1211 Geneva, Switzerland; 2 Computer Science Department, University of Geneva, Geneva, Switzerland; 3 EA 7394-ICV- Institut Charles Viollette, University of Lille, INRA, ISA, University of Artois, Univ. Littoral Côte d’Opale, 59000 Lille, France; 4 UMR 9189- CRIStAL- Centre de Recherche en Informatique Signal et Automatique de Lille, University of Lille, CNRS, Centrale Lille, 59000 Lille, France; 5 Bonsai Team, Inria-Lille Nord Europe, 9655 Villeneuve d’Ascq Cedex, France; 6 Vital-IT Group, SIB Swiss Institute of Bioinformatics, Amphipole Building, Quartier Sorge, 1015 Lausanne, Switzerland; 7 Section of Biology, University of Geneva, Geneva, Switzerland

**Keywords:** Peptide, Monomer, Retro-biosynthesis, Fragmentation, Structure analysis, Natural product, Curation, Substructure search

## Abstract

Proteinogenic and non-proteinogenic amino acids, fatty acids or glycans are some of the main building blocks of nonribsosomal peptides (NRPs) and as such may give insight into the origin, biosynthesis and bioactivities of their constitutive peptides. Hence, the structural representation of NRPs using monomers provides a biologically interesting skeleton of these secondary metabolites. Databases dedicated to NRPs such as Norine, already integrate monomer-based annotations in order to facilitate the development of structural analysis tools. In this paper, we present rBAN (retro-biosynthetic analysis of nonribosomal peptides), a new computational tool designed to predict the monomeric graph of NRPs from their atomic structure in SMILES format. This prediction is achieved through the “in silico” fragmentation of a chemical structure and matching the resulting fragments against the monomers of Norine for identification. Structures containing monomers not yet recorded in Norine, are processed in a “discovery mode” that uses the RESTful service from PubChem to search the unidentified substructures and suggest new monomers. rBAN was integrated in a pipeline for the curation of Norine data in which it was used to check the correspondence between the monomeric graphs annotated in Norine and SMILES-predicted graphs. The process concluded with the validation of the 97.26% of the records in Norine, a two-fold extension of its SMILES data and the introduction of 11 new monomers suggested in the discovery mode. The accuracy, robustness and high-performance of rBAN were demonstrated in benchmarking it against other tools with the same functionality: Smiles2Monomers and GRAPE.

**Electronic supplementary material:**

The online version of this article (10.1186/s13321-019-0335-x) contains supplementary material, which is available to authorized users.

## Introduction

Natural products are a well-recognized source for drug discovery due to their wide range of antibiotic, antitumor or immunosuppressant activities. Indeed, 26% of the drugs approved by the US FDA from 1981 to 2014 were natural products or natural products derivatives [1]. An important part of those are nonribosomal peptides (NRPs) considered as *secondary metabolites* and found in bacteria and fungi. In these organisms, NRPs are assembled by large enzymatic systems into complex structures from building blocks such as non-proteinogenic amino acids, fatty acids or carbohydrates. Significant portions of the bacterial and fungal genome are devoted to the production of these compounds. Therefore, genome mining tools such as GARLIC [2] and antiSMASH [3] have been developed to automatically identify secondary metabolite biosynthesis gene clusters. However, these tools are not able to distinguish between clusters of already known compounds and clusters uncovering new natural products. A possible approach to solve this problem is to perform the retro-biosynthesis of these compounds obtaining their constituent monomers and align them with the monomers of the predicted clusters [2, 4, 5]. A few methods predicting the retrosynthesis of a compound from its chemical structure have been described. To begin with, CHUCKLES [6] can convert a chemical structure into a monomer-based sequence by matching a set of monomers against the target structure. The monomers are previously sorted by descending size and the matching is done sequentially. The main limitations of this method are: (i) larger monomers are given the priority and (ii) monomers with more than three external connections are not handled. This approach is efficient with regular peptides, but not for NRPs. Other methods such as RECAP (Retrosynthetic Combinatorial Analysis Procedure) [7], BRICS (Breaking retrosynthetically interesting chemical substructures) [8] or molBLOCKS [9] use fragmentation rules to obtain drug-like chemical entities. However, these methods are focused on the discovery of structural motifs for drug design and they make no attempt to annotate the target compounds by identifying the resulting fragments. Moreover, their fragmentation rules are derived from common chemical reactions, lacking specificity for particular compounds such as NRPs.

In recent years, two new tools specifically designed to target NRPs have been published. The first one, Smiles2Monomers (s2m) [10] maps the monomers of a database within an atomic structure and selects the best combination (tiling) that covers the whole molecule with non-overlapping monomers. This approach is algorithmically elegant but computationally expensive. As a result, the best tiling is obtained as an approximate solution and the optimal mapping is not always found, sometimes leading to uncovered regions in the molecule. A second solution is implemented in GRAPE (Generalized Retro-biosynthetic Assembly Prediction Engine) [2] as the theoretical deconstruction of NRPs and Polyketides (PKs) by applying specific retro-biosynthetic reactions. The obtained fragments are then matched against a monomer library integrated in the software. A sequence of monomers is given as a result, but the original monomer linkages are lost. Both, GRAPE and s2m rely on their monomer database, which is a limitation for the analysis of peptides containing new monomers.

Part of the interest in developing retro-biosynthesis tools arises from the benefit of a monomeric representation. Chemical structure databases dedicate an important part of their resources in data curation, analysis and visualization. The complex structure of NRPs often results in too dense and unclear atomic representations. A monomeric format, as with peptide sequence annotation, reduces the complexity of the layout providing the same information in a more intelligible way and facilitates the implementation of substructure and similarity search algorithms [11, 12]. Furthermore, this format is biologically meaningful as the monomers provide direct insights into the peptide activity and origin [11, 13, 14]. These substructures bring essential information to understand the biosynthesis of the peptide and, given their bioactivities, they are interesting data for structure-based drug design studies.

The convenience of the monomeric method is reflected in the emergence of new monomer-based notation formats. Examples of that are the recent languages named HELM (Hierarchical Editing Language for Macromolecules) [15, 16] and SCSR (Self-Contained Sequence Representation) [17], which provide concise annotation of complex biopolymers in a component-based approach. Some databases devoted to bioactive peptides have also chosen this format to represent their data. This is the case of Norine [18, 19], which is entirely dedicated to NRPs and uses monomer graphs for structure depiction and analysis. Indeed, all the structural analysis tools integrated in Norine are monomer-based [10–12, 14], proving the advantages of the approach. Another example is the BIRD (Biologically Interesting molecule Reference Dictionary) [20] project from PDB (Protein Data Bank) [21]. Here, the peptide-like inhibitor and antibiotic molecules are represented as polymers with sequence information or as single components. BIRD is the result of a remediation work in which part of the PDB entries were reviewed in order to improve their representation and facilitate their identification and analysis. This kind of processes require a long and tedious effort that could be accelerated using bioinformatics tools. Hence, the usage of retro-biosynthesis software is decisive to improve these curation tasks by providing automatic annotation and assuring conciseness between the atomic and monomeric annotations. Additionally, the “in silico” retro-biosynthesis can also be applied to validate already annotated entries by checking the correspondence between the existing and the predicted annotations. A practice that would also spot potentially erroneous entries.

**Fig. 1:**
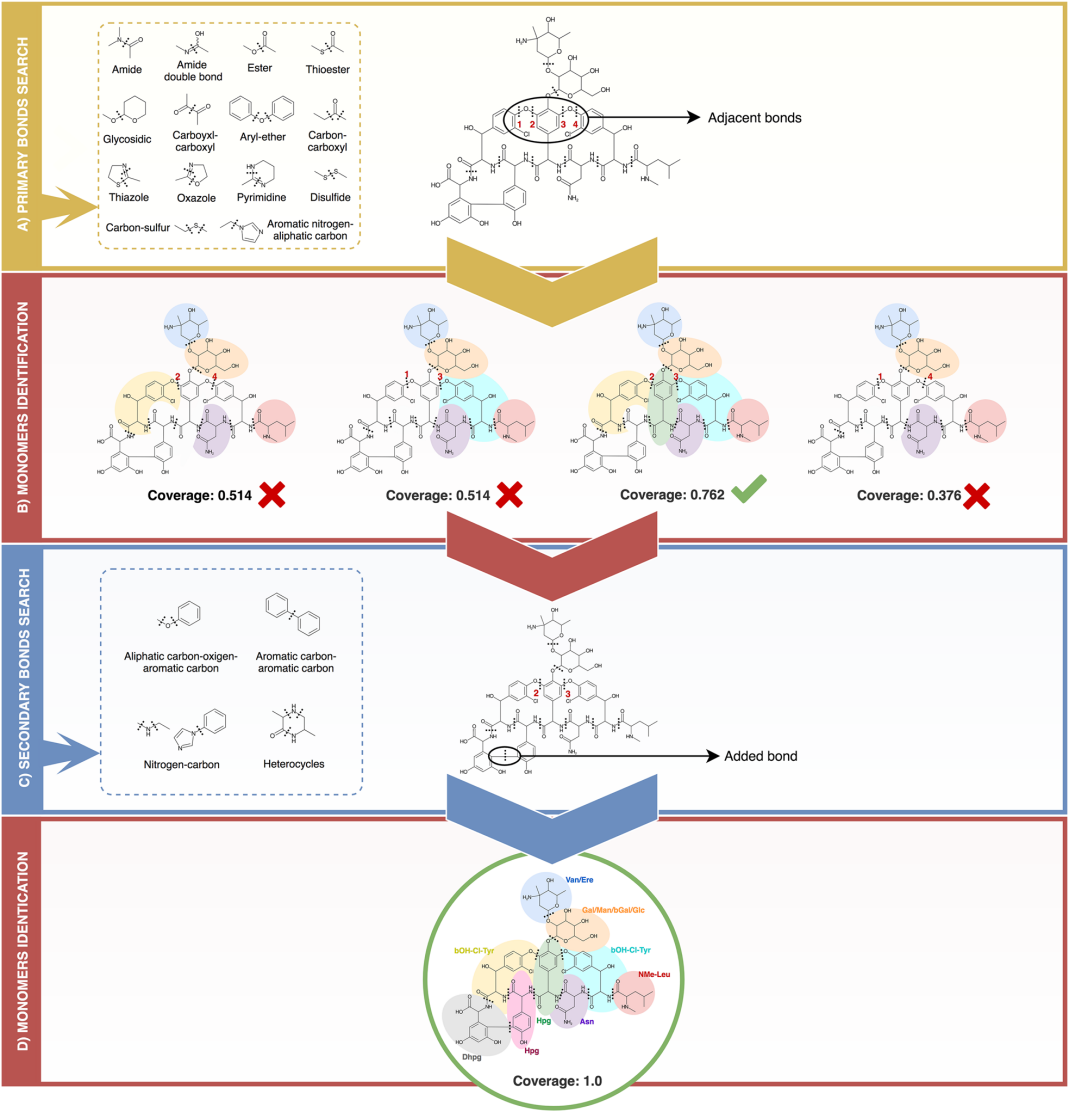
Example of Vancomycin processing. **A** First, the primary bonds mapping searches the most common bonds between NRP monomers within the molecule. This process results in the mapping of two pairs of adjacent bonds that cannot be targeted simultaneously since it would isolate some atoms. To avoid that all the possible combinations only including one of the neighboring bonds are computed. **B** Then, rBAN retrieves the substructures resulting from each combination and it matches them against the monomer database. A coverage score is given to each combination based on the number of atoms that could be annotated. **C** In this case, any of the results has a full coverage, so the algorithm proceeds to the secondary bonds search of the structure with the highest score. **D** The breakage of a carbon-carbon bond results in the full mapping of the peptide

In this article, we introduce rBAN, a new tool simulating the retro-biosynthesis of NRPs. The main strategy of the software is to perform the fragmentation of a molecule by breaking it through a set of pattern bonds and matching the resulting fragments to a monomer database (Fig. 1). The matching process was specifically designed to allow tautomer’s identification, a feature that was already presented in the s2m tool and named light matching. However, the two approaches are slightly different: the light matching of s2m omits all the implicit hydrogens and bond orders to match the monomer, while rBAN only omits the position of the double/triple bonds, making the method more restrictive and decreasing the likelihood of obtaining false positives. rBAN also introduces the “discovery mode” option that is applied when a monomer cannot be matched in the custom database. In this mode, missing substructure(s) can be automatically searched in PubChem [22] to suggest a new monomer. This feature reduces the dependence to the database, providing more flexibility than the retro-biosynthetic approaches previously presented. Finally, the results are presented in a directed graph format that includes the bond types linking the monomers. To our knowledge, no other tool provides the bond type annotation though it can be highly relevant for its integration into structural analysis pipelines. rBAN is presented in two formats, as an executable jar and as a web application, the latter being a simplified version of the software. We used rBAN for the curation of the Norine database and benchmarked it against s2m and GRAPE.

## Methods

**Fig. 2:**
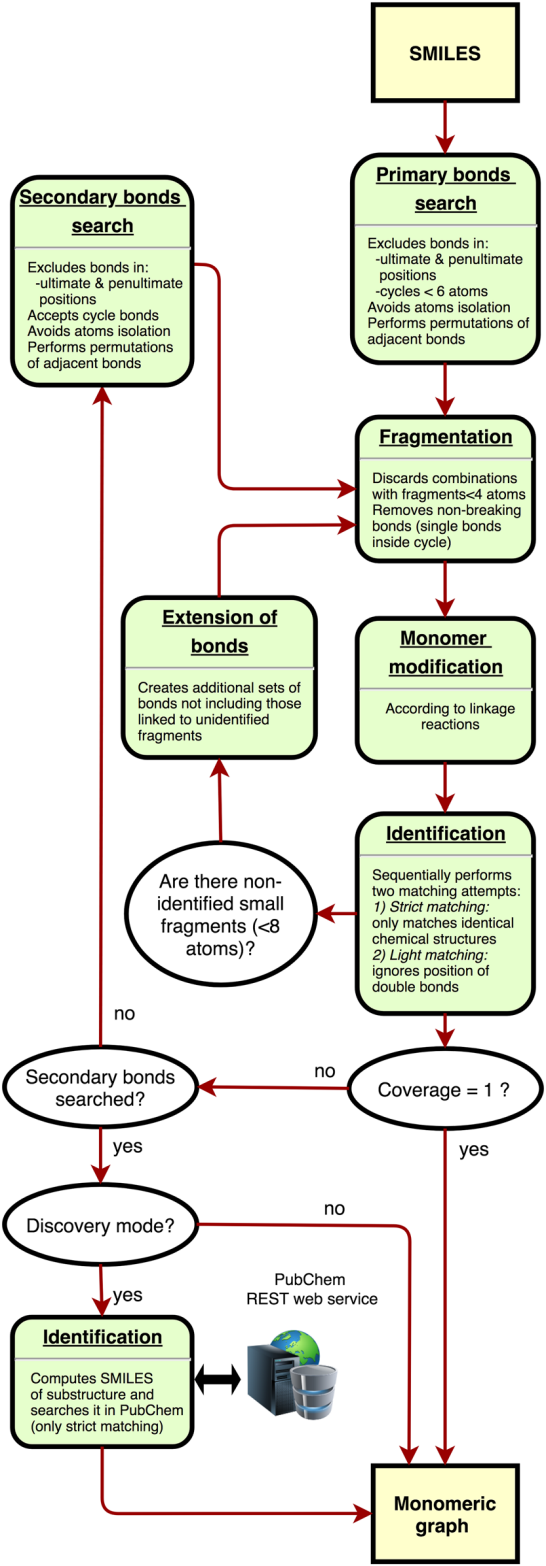
Software architecture workflow. This flowchart describes the series of steps for processing structures with rBAN

rBAN was developed in Java using the Chemistry Development Kit (CDK) library. Given an input file with chemical structures in SMILES (Simplified molecular-input line-entry system) [23], the tool uses CDK to map the target bonds by substructure search and Norine monomer database to identify the corresponding monomers. The overall process of the software architecture is described in the Fig. 2.

### Data preprocessing

#### NRPs monomer data

Norine is dedicated to NRPs and was used to retrieve the monomer dataset in order to guarantee consistency between the target compounds and their building blocks. This dataset consists of 534 manually-annotated monomers extracted from the compositions of the NRPs in the database. Hence, the dataset is limited to the monomers present in Norine peptides and it may not be sufficient when used for the identification of fragments of new NRPs. To solve this issue, we developed an algorithm that suggests new monomers by adding modifications to the existing ones. In order to add a biological value to the predicted structures, the modifications were selected in accordance to some of the enzymatic reactions occurring in the NRP biosynthesis [24, 25] (see Additional file 1: Table S1). For instance, a methyl group is added in the amino side of each monomer in order to mimic the action of the methyltransferase (MT) domain. Finally, preprocessing is also used to identify monomers with identical chemical graphs (isomers) and group them as a single entry (the tool does not include isomer discrimination). The PubChem PUG-REST service is used to include the PubChem IDs of the monomers.

### Software architecture

1. *Primary bond search* NRP monomers are usually connected through certain types of bonds, the most common being amino and ester. Therefore, mapping these bonds is the first step of monomer identification. We rely on a graph isomorphism algorithm provided by CDK to search the substructures of the bonds within the chemical graph of the target compound. The complete list of bond types included in the search (Fig. 1A) was manually constructed based on observations and literature [25–27]. Smiles Arbitrary Target Specification (SMARTS) [28] is the language used to describe the molecular patterns of the bonds since it provides higher flexibility than SMILES. During this step, all the bonds matching the target patterns will be selected unless they are positioned on terminal branches of the chemical structure (ultimate or penultimate positions). These bonds are excluded in order to avoid single atom isolation. Bonds pertaining to cycles of less than six atoms are also removed, although they will be evaluated later in the pipeline. The only exception to that rule are the oxazole and thiazole heterocycles, as they are highly abundant in NRPs [29].
  Once mapped, the bonds between adjacent positions cannot be simultaneously targeted (single atom isolation problem). This issue is solved by computing multiple combinations, each combination only including one of the neighboring bonds. To do so, the adjacent bonds are grouped in different sets and a recursive algorithm computes the Cartesian product of these sets to generate all possible permutations. Note that to reduce the number of combinations and maximize the number of targeted bonds not all the adjacent bonds are included in this calculation, but only those whose simultaneous breakage implies the isolation of single atoms or pairs (Fig. 3). In a similar way, the presence of an amino or an ester bond in the set also limits combinatorics as they are prioritized due to their predominance as NRP links.

**Fig. 3:**
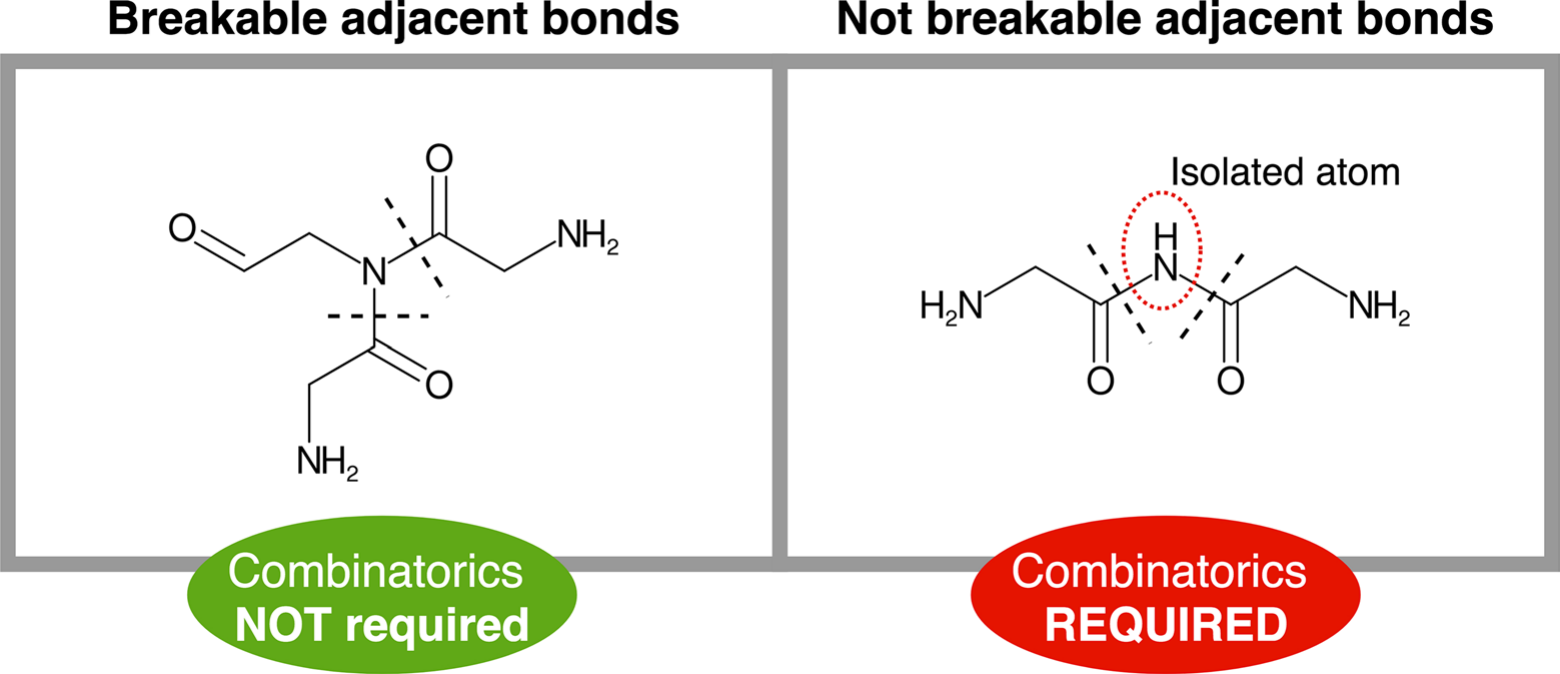
Adjacent bonds breakage. Our fragmentation algorithm avoids atom isolation, which restricts the simultaneous cut of some adjacent bonds, requiring the computation of further combinations
2. *Fragmentation and identification* The bonds mapped in the primary search are used as breaking points to obtain the fragments of the molecule. This is done using a breadth-first search algorithm to iterate through the chemical graph and compute the resulting fragments from those breakages. This action is performed for each permutation of bonds provided, producing several sets of fragments that will be matched against the monomer database. Prior to this matching, the fragments are slightly modified in order to compute their expected structure outside the molecule –when not linked- thereby generating structures equivalent to those stored in the monomer database. The modifications applied are in accordance with the linkage patterns observed for each type of bond. For instance, a hydroxyl group is added to the formyl-ended fragment derived from a peptide bond breakage in order to obtain the “original” carboxyl-terminus structure of the monomer (see Additional file 1: Table S2). Once these modifications are applied, the monomers are matched against the database in order to identify them. Two different matching attempts are sequentially executed: the strict and the light matching. The strict matching will be only successful if the graph of the fragment is identical to the graph from the database. It checks the atom connectivities, the atom types and the bond orders. If a structure cannot be “strictly” matched, rBAN proceeds to light matching, which allows changes in the position of the double/triple bonds facilitating tautomer identification. Failure to match fragments can be due to the fragmentation of inner bonds in a monomer. Hence, when a fragment is not identified, the algorithm repeats the matching process by removing each of its linking bonds consecutively (Fig. 4). This process is limited to small-medium fragments (less than 8 atoms) because of their higher chances of being part of a monomer; such restriction also avoids an excessive amount of combinations. When a whole set of fragments has been matched, it is assigned with a score indicating the number of annotated atoms versus the total number of atoms in the molecule (coverage). The next steps in the pipeline depend on these scores. If any of the fragment sets has a score of 1, ergo all the monomers have been identified, the algorithm proceeds to the monomer graph creation. Otherwise, the secondary bonds search is applied to the sets with the highest score (Fig. 1B).

**Fig. 4:**
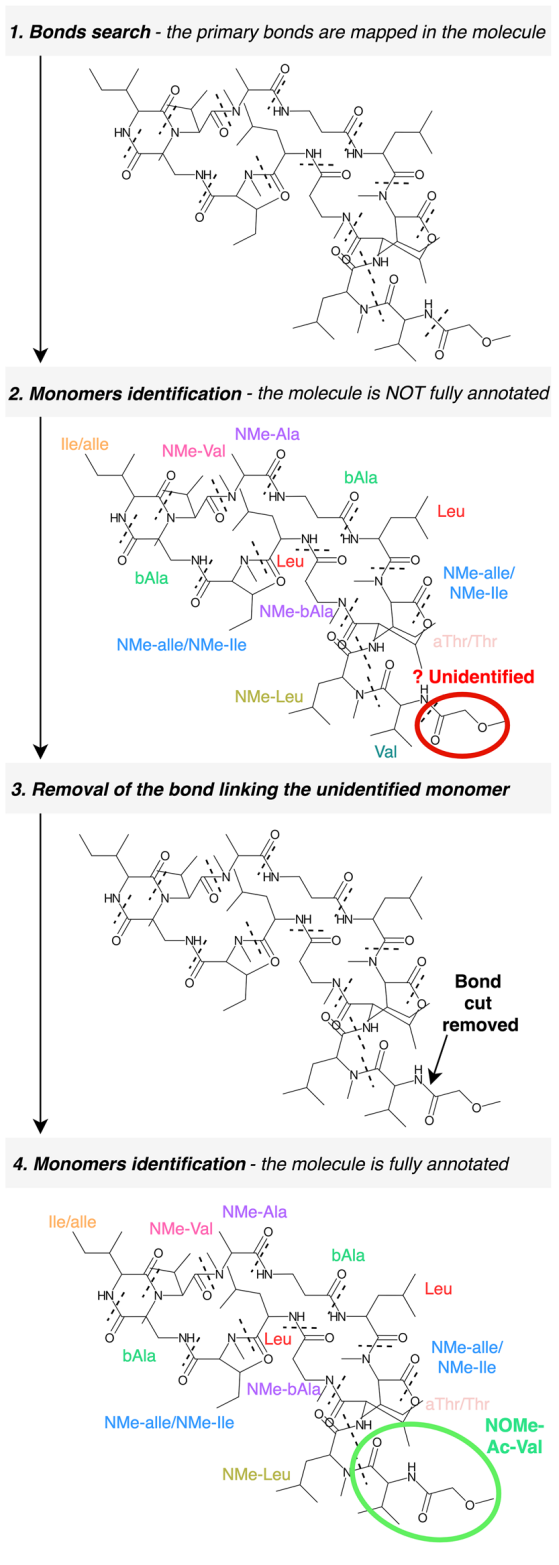
Identification of monomers containing inner bonds. Some monomer bonds are sometimes fragmented by the algorithm. To handle these cases, when a small region cannot be identified, rBAN repeats the matching process after removing the bond linked to the unidentified substructure (example with *Theonellapeptolide* Ie)
3. *Secondary bond search* Some bond types such as the carbon-carbon linkages are not common as a bridge between NRP monomers and breaking them in the initial step would lead to unnecessary and excessive fragmentation. This is why they are considered as secondary bonds and their mapping is restricted to the fragments that have not been identified. The secondary bond collection comprises less common bonds and non-specific heterocycles (Fig. 1C). Specific heterocycles such as the oxazoles and thiazoles are covered in the primary search, since their cyclisation patterns are well-known [27, 30]. Yet the existence of a wide range of cyclisation forms complicates the individual targeting of the remaining heterocycles. For this reason, we use a general approach that provides several breakage possibilities instead of a single solution. The algorithm performing this task implements substructure search to identify the heterocycles and combinatorics to return the permutations of cycle bonds that break the fragment without leaving isolated atoms. After the secondary bond search, the fragmentation and identification step is repeated. If the full score is still not reached and the monomer discovery mode is activated, rBAN moves to the next step.
4. *Monomer discovery* The unidentified substructures may represent missing monomers in the database. In these cases, the CDK library is used to generate the SMILES of the unknown chemical structure that serves as a parameter for an automatic PubChem search. The substructures successfully identified are annotated using their PubChem name and suggested in the results as new monomers for the Norine database. The information is presented in a JSON file where the compounds containing the suggested monomer are also listed. Graphical results are also provided. For each new monomer, rBAN creates a folder with the depictions of the peptides where the substructure occurs.
5. *Monomer graph serialization* The monomeric structure consists of a directed graph with a set of nodes represented by the predicted fragments and a set of edges symbolizing their linking bonds. To build this graph, the monomers are reconnected using the association between the broken bonds and the resulting fragments. The edges are labeled specifying the type of bond and their direction is chosen based on the type of atoms in the bond. The monomer associated with the carbon atom of the bond is set as the source while the monomer containing the heteroatom is set as the target. For instance, in case of a peptide bond, the monomer with the carboxylic side would be the source while the monomer with the amino side, the target. The graph is serialized in a JSON file also containing the atomic graph of the peptide that associates each atom with the monomer containing it (see Additional file 2). If the theoretical annotation (Norine graph) is given as an input, the output graph will also contain a “correctness” value. This value results from the division of the number of correctly annotated atoms (associated with the expected monomer) by the total number of atoms in the molecule. The graphical depiction of the chemical structures with the labeled monomers is also implemented as an option.

## Results and discussion

The Norine database provides structural data of NRPs in both atomic and monomeric formats. The monomer annotation is essential to obtain the correctness of rBAN predictions and for this reason Norine was chosen as the main resource to evaluate the software.

### Norine Database Curation and Extension

**Fig. 5:**
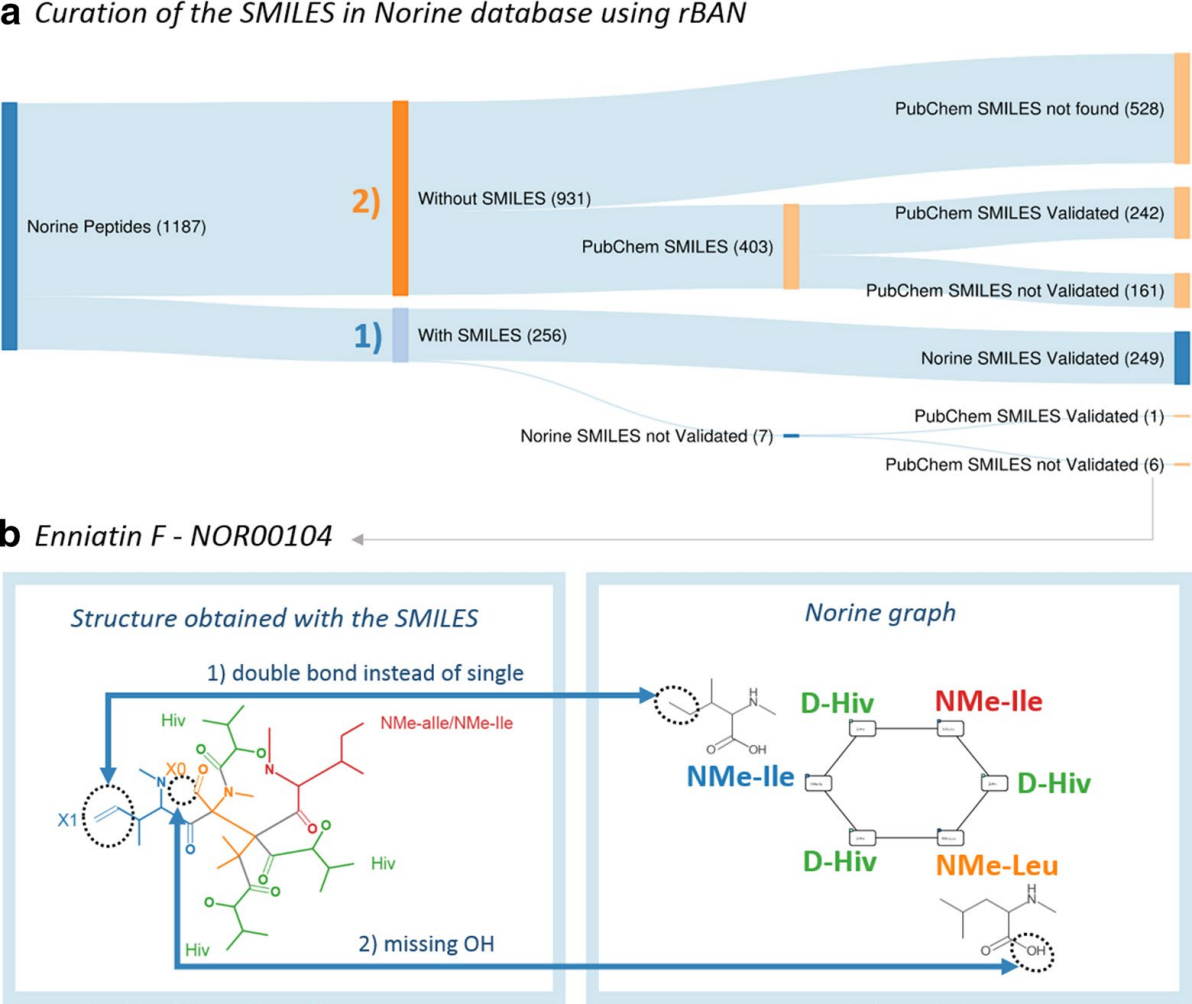
Norine curation. **a** The curation involves two main steps: (1) Automatic verification and correction of the SMILES in Norine. rBAN validated 249 (97.26%) SMILES and identified seven potential erroneous SMILES. Retrieving the PubChem SMILES from the non-validated entries enabled the correction of the SMILES of Motuporin (NOR00825). The manual inspection of the remaining entries concluded with the confirmation of six wrong SMILES. (2) Automatic addition of SMILES retrieved from PubChem. From the 403 SMILES retrieved from PubChem, 242 were validated using rBAN. The 161 not validated are likely to be false positives due to the ambiguity of the PubChem searches performed. **b** Enniatin F belongs to the set of non-validated peptides. rBAN failed to validate this peptide due to differences between the molecular and monomeric annotations. The monomeric graph is circular and contains *N*-Methyl-Isoleucine while the SMILES encodes a linear structure with dehydro-*N*-Methyl-Isoleucine(1). Additionally, rBAN could not identify what is supposed to be a *N*-Methyl-Leucine because it misses a hydroxyl group (2)

In Norine, the SMILES (atomic structure) and the monomer graphs (monomeric structure) are sometimes extracted from different resources. To guarantee the conciseness between the two representations and thereby validating the SMILES from Norine, rBAN was run to compare the SMILES-predicted monomeric graph with the Norine annotated graph. When the theoretical and the predicted graphs are identical, then the result is considered as correct and the corresponding SMILES is validated. From the 256 peptides that are described in SMILES, rBAN could validate 249 (97.26%) (Fig. 5a1). The non-validated peptides were manually inspected and errors in their SMILES were identified. Hence, the lack of validation was attributed to wrong input data and not to a wrong mapping of the software. In fact, the software helped to spot these wrong SMILES that were later corrected/removed from the database. An example is Enniatin F, whose monomeric annotation did not match the structure given by the SMILES (Fig. 5b).

As already mentioned, in the previous version of Norine only 256 entries (21.56% of the total) contained the structural information in the SMILES format. In order to increase this count, we used the PubChem PUG-REST Service to perform automatic searches, retrieve the missing SMILES and validate them using rBAN. The only available parameters for the PubChem searches were the name of the compound, which lacks specificity, and the PubChem link provided by Norine, that is rarely present and occasionally wrong. Hence, the validation step becomes essential to reduce the uncertainty of the search and provide more reliable results. From the 403 SMILES retrieved from PubChem, 242 were validated using rBAN (Fig. 5a2). These SMILES were added to the database generating a two-fold increase in Norine SMILES data. The non-validated entries were considered as false positives due to the uncertainty of the search.

### Monomers discovery

As already mentioned, the non-validated entries can be due to a wrong annotation either in the SMILES or in the monomeric graph. In the latter case, peptides may contain monomers not present or wrongly annotated in Norine. Thus, rBAN was run in discovery mode to identify new monomers. The software suggested 61 new building blocks. Some of these predictions could be wrong due to mistakes in the input SMILES or wrong mapping of the software. Hence, a manual inspection was required before their addition into the database. To increase confidence, only the monomers present in more than one compound were evaluated.

From the 18 monomers examined, eleven were correct suggestions (Table 1). *N*-Formyl-Lysine was the most commonly found monomer, missing in Norine because CO is currently defined as a monomer in the database and occurs in several NRP graphs. In contrast, rBAN considers CO as formylation and not a monomer therefore suggested a new formylated monomer. Most of the other new entities correspond to monomers that were not properly annotated in the monomeric graph. Such is the case of the ”C4:1(3)–OH(2)” monomer that should be beta-Vinyllactic acid (C5:1(4)–OH(2)) (see Additional file 1: Fig. S1). Other cases encompass a missing monomer in the monomeric graph or an incorrect SMILES of the known monomer. All the corrections were made in accordance to the literature associated with the corresponding compounds.

**Table 1:** Monomers correctly suggested by rBAN

Norine code	PubChemID	IUPAC name	Structure	Compounds	Reason of the missing monomer	Refs.
NFo-Lys	12679627	6-amino-2-formamidohexanoic acid		NOR00261, NOR00262, NOR00263, NOR00264 NOR00266, NOR00267, NOR00269, NOR00270 NOR00271, NOR00272, NOR00274, NOR00275 NOR00276, NOR00277, NOR00278, NOR00580	“CO” monomer in graphs	[32]
D-3OMe-Ala	97963	2-amino-3-methoxypropanoic acid		NOR00422, NOR00423, NOR00424, NOR00425 NOR00588	Wrong SMILES of D-3OMe-Ala monomer	[33]
C5:1(4)-OH(2)	172026	2-hydroxypent-4-enoic acid		NOR00064, NOR00066, NOR00068, NOR00071 NOR00073	Wrong monomer in graphs: C4:1(3)-OH(2) -> C5:1(4)-OH(2)	[34]
N-Suc	12522	4-amino-4-oxobutanoic acid		NOR00160,NOR00166, NOR00903	Missing monomer in graphs	[35, 36]
C5:0-OH(2)-Ep(4)	54305979	2-hydroxy-3-(oxiran-2-yl)propanoic acid		NOR00086, NOR00087	Wrong monomer in graphs: C4:0-OH(2)-Ep(3) -> C5:0-OH(2)-Ep(4)	[34]
Gen	3469	2,5-dihydroxybenzoic acid		NOR00489, NOR00598	Wrong monomer in graphs: 2,3-diOH-Bz -> Gen	[37, 38]
C10:0-OH(2)-NH2(3)	57484230	3-amino-2-hydroxydecanoic acid		NOR01134, NOR01135	Wrong monomer in graphs: Adda -> C10:0-OH(2)-NH2(3)	[39]
iC6:0-OH(2.4)	55300467	2,4-dihydroxy-4-methylpentanoic acid		NOR00078, NOR00077	Wrong monomer in graphs: iC5:0-OH(2.3) -> iC6:0-OH(2.4)	[34]
Isovaleric_acid	10430	3-methylbutanoic acid		NOR00477	Wrong monomer in graph: Hiv -> Isovaleric_acid	[40]
D-Cl-Trp	65259	2-amino-3-(6-chloro-1H-indol-3-yl)propanoic acid		NOR00554	Wrong SMILES of D-Cl-Trp monomer	[41]

Among the suggested monomers, *N*-Formyl-Lysine is the most abundant. rBAN considers CO as a formylation, therefore suggests a new formylated monomer instead of using the “CO” monomer currently present in Norine. A second new entity present in five compounds is D-3OMe-Ala. In this case the monomer name is correct but not the SMILES associated with it. Most of the other suggestions are due to the monomers wrongly annotated in the graph that should be substituted with a new substructure. There is also one case (N-Suc) where the monomer was directly missing in the graph. All these corrections were manually evaluated to confirm the agreement with the literature

Seven of the monomers suggested by rBAN were rejected (find them in Additional file 1: Fig. S3) because the manual inspection of their corresponding peptides revealed that their SMILES rather than their monomeric graph created the problem. The peptidic structures (SMILES) of these records contained errors or did not even correspond to the right molecule due to the ambiguous PubChem searches previously performed.

The eleven new monomers were added in the Norine database along with the correction of the wrong annotations, either in the monomeric graphs or in the SMILES of the compounds (find examples in Additional file 1: Fig. S1). This step was essential for evaluating the increase of the validated data. In the end, 11 added new monomers along with the correction of wrong annotations boosted the count from 492 to 526 validated entries.

### Benchmarking

rBAN was compared against two tools with similar functionality: s2m and GRAPE. The benchmarking was performed on a PC computer with an Intel/Core i5-5300U CPU at 2.3 GHz with 4 GB of RAM allocated to the Java Virtual Machine.

#### rBAN vs s2m

Within the retro-biosynthetic tools targeting NRPs, s2m is the closest to rBAN as it produces the same output: a monomeric graph. Yet the two approaches substantially differ in their features and algorithmic approaches set to handle the issues raised by mapping the molecules. These involve among others, the monomer search, the light matching or the heterocycles treatment (see Table 2). In order to compare both tools, we analyzed their results, their robustness and their computational performance. The benchmark in the following sections was performed running s2m in the light matching mode to allow tautomer identification and obtain results comparable to those of rBAN.

**Table 2:** Comparison rBAN versus s2m

	rBAN	Smiles2Monomers
a) Monomers mapping	Based on molecule fragmentation through common monomer linking bonds	Based on mapping of monomers and selection of best tiling
b) Light matching	Positions of double/triple bonds are ignored	Implicit hydrogens and bond order are ignored
c) Heterocycles treatment	Accounts for NRP cyclisation patterns initiating oxazoles and thiasoles formation	Does not include any rule/pattern for heterocycles
d) Presence of new monomers	Unmatched regions left unannotated and potentially identified in discovery mode	Matches the most similar monomers in a given database and leaves out uncovered atoms
e) Graph serialization	Labelled edges with bond type and directed in accordance to functional groups in each side	Unlabelled edges

a) To map the monomers rBAN fragments the molecule and matches the results against the monomer database. S2m computes the combinations of monomers that fit in the molecule. b) To enable tautomer identification during the matching process rBAN omits the positions of the double bonds in the monomer, but it keeps considering those, becoming more restrictive than its analog mode in s2m, in which neither the implicit hydrogens nor the bonds order are taken into account. c) Characteristic NRP structural patterns such as heterocycles are specifically targeted in rBAN but not in s2m. d) When a region cannot be matched because of the absence of the monomer in the database, rBAN leaves the whole region unannotated (with the option of recurring to the discovery mode), while s2m tries to match the most similar monomer even if this is a wrong match and it implies leaving unannotated atoms. e) The monomers graph from rBAN has the edges labeled specifying the type of bond and its direction. s2m does not provide bond labels

##### Results comparison

**Fig. 6:**
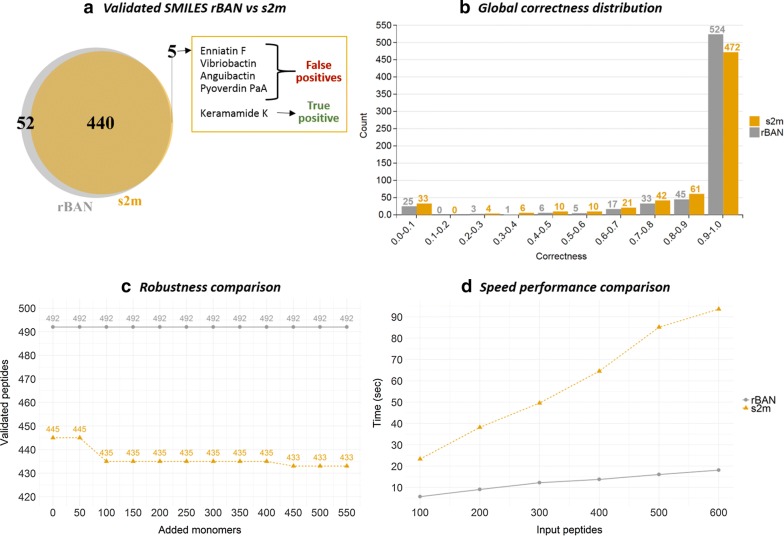
Benchmarking rBAN versus s2m. **a** Both software were used to validate the SMILES data by comparing the Norine monomer graphs with the SMILES-based predicted graphs. rBAN could validate more peptides than s2m and four of the entries uniquely validated by s2m turned out to be false positives of the software. The manual examination of the entries uniquely validated by rBAN revealed a better capacity of the tool to annotate large structures and peptides containing heterocycles and tautomers. **b** The global distribution of the correctness do not show substantial differences between the two software but it proves that rBAN does not only have more correct peptides, but also less peptides with correctness values close to zero. **c** The monomer database was extended with new chemical entities to evaluate its effects on the peptide mapping. The results of rBAN remained unchanged proving its robustness, while the extension of the monomer database affected mapping in s2m. **d** The computational performance was evaluated with different amounts of input peptides. In all cases rBAN outperformed s2m, being between four and five times faster

s2m was run to validate the same SMILES data previously used in the curation protocol of the Norine database. Out of the 659 peptidic structures retrieved from Norine and PubChem, s2m validated 445. Although the same process with rBAN resulted in a higher amount of validations (492), the comparison singled out five entries that were only verified by s2m (Fig. 6a). These entries were reviewed to identify the reasons why rBAN could not validate them . However, manual inspection only confirmed the validity of a single record, as the rest was not properly matching their monomeric counterparts and turned out to be false positives of s2m. Among these structures, Ennitatin F (Fig. 5b) that was reported earlier as cyclic and containing NMe-Leucine and NMe-Isoleucine. s2m maps these monomers in the structure yet the NMe-Leucine is missing a hydroxyl group while the NMe-Isoleucine has an additional double bond. These artefacts are related to the method of precomputation and light matching in s2m. Prior to the analysis, the precomputation of s2m generates for each monomer all the possible residues that may occur due to the loss of functional groups during the linkage with other monomers. These residues are the substructures that will be mapped by the software to identify the monomers. This strategy loses the association between the linkage and the loss of the functional group. That leads to wrong matches when the implicit hydrogens are not considered (as set in the light matching mode). This is the case of NMe-Leucine that is matched although it misses the hydroxyl group of the carboxyl end, which would be the expected structure if it was linked to another monomer, but is wrong when it is terminal in the molecule (see Additional file 1: Fig. S2). The three other false positives of s2m show similar problems. The fifth entry is Kermamide K, the only true positive in the set. It was not validated by rBAN because this software does not consider CO as a monomer.

The manual evaluation of the 52 peptides uniquely validated by rBAN confirmed their validity and uncovered some structural patterns that were optimally handled by rBAN and not by s2m. The fragmentation model implemented in rBAN properly annotated large peptide structures whose monomeric composition was not revealed by the tiling algorithm of s2m. Similarly, the annotation of NRPs containing thiazoles and oxazole heterocycles was successfully carried out using rBAN, while the results of s2m did not match the monomer graph. Another pattern also observed in the rBAN-validated entries was the presence of monomers whose hydrated and dehydrated forms coexist in the monomer database. The restrictive light matching of rBAN succeeded in distinguishing them, while the light matching approach of s2m led to wrong monomer assignments. Finally, to complete the picture of correctness, we computed the distribution of correctness values from each software (Fig. 6b). Both tools showed a similar distribution though slightly shifted. rBAN generates more highly scored peptides (0.9–1) and less with correctness close to 0.

##### Robustness comparison

The existence of several combinations of monomers mapping the same peptide substructure increases the complexity of the problem. Hence, the extension of the monomer database can easily influence the mapping of a molecule and could lead to the appearance of wrong annotations that were previously correct. The robustness of the two software was tested while extending the monomer database and evaluating its impact on the results. An additional set of monomers was obtained using the PubChem Classification browser to retrieve the chemical entities defined as non-proteinogenic amino acids (ChEBI Ontology). Components with a molecular mass higher than 450 g/mol were discarded, as they greatly exceeded the average monomer size. Chemical structures already present in the monomer database were also discarded to avoid repetitions. A total of 550 monomers were sequentially added in order to test the response of both software to different extensions of the database (Fig. 6c). rBAN correctly annotated the same amount of entries (492) for all the database sizes. Note that the number of correct annotations could not be improved because the Norine graphs were not modified to include the new monomers so maintaining the same correctness was the best that could be expected, proving the robustness of the software. On the other hand, s2m correct results dropped from 445 to 435 with the addition of 100 new monomers, although the rest of the extensions was steadily handled, only dropping by two in the extension to 450 monomers.

##### Computational performance comparison

For the evaluation of the computational performance, the timing was limited to the analysis and did not account for the generation of images. We registered the performance of each software varying the number of input peptides from 100 to 600. To obtain the average performance each measurement was repeated five times. rBAN was significantly faster than s2m (Fig. 6d). As expected, computing time increased with the number of peptides and the difference between the two software remained 4 and 5-fold. Although this trend is likely to be confirmed, these measurements may change with a different set of peptides, as computing time depends on the complexity of the chemical structures analyzed. Note that with rBAN, using the discovery mode feature would also change the performance results as the computation time increases due to the RESTful HTTP requests performed to retrieve data from PubChem.

#### rBAN vs GRAPE

**Fig. 7:**
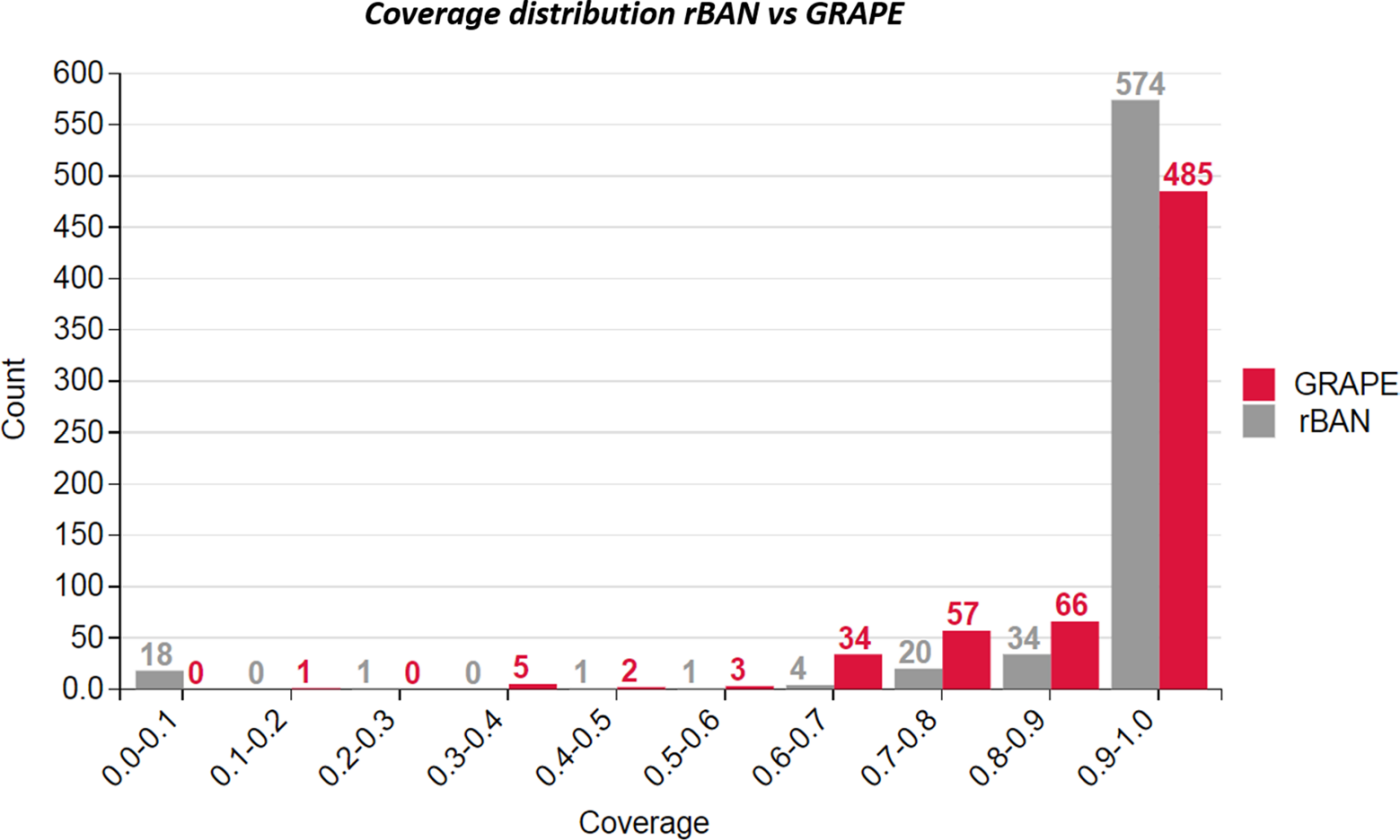
Benchmarking rBAN versus GRAPE. The coverage of the annotations given by each software was compared. The distribution shows that rBAN fully annotated more peptides than GRAPE

As already mentioned, GRAPE is another tool for the retro-biosynthesis of NRPs and polyketides (PKs). However, the annotations provided by this software differ from those of rBAN as (1) they are based on a different monomer library and (2) the modifications are annotated separately from the monomers. These differences make the comparison of correctness difficult and that explains why the benchmark was limited to the analysis of coverage (ratio between annotated atoms and total number of atoms in the molecule). The same set of SMILES without the wrong entries previously identified  was used to test GRAPE. Out of 653 peptide structures, GRAPE fully annotated 468, while rBAN reaches 560 annotated entries, 492 of them being correct. In fact, from these results it is possible to indirectly compare the correctness of the two software. Only the peptides with a full coverage can have full correctness. Hence, assuming that all the annotations from GRAPE are correct (468), the result is still lower than the number of correctly annotated peptides from rBAN (492). The whole distribution of coverage shows how GRAPE tends to leave less peptides with low coverage (Fig. 7). Nevertheless, the annotations of the 18 peptides with zero coverage in rBAN were manually checked for GRAPE. As it turned out, their monomer fragments were categorized as “unknown”. Finally, the computational performance was evaluated using the same data. rBAN analysed the 653 peptides in an average time of 26.94 s, while the same process with GRAPE resulted in an average time of 81.34 min.

##### Web implementation

A web application interface was designed and integrated into Norine as an additional tool for the database curation. With the aim of providing a simple and user-friendly interface, the online version of rBAN is limited to the analysis of a single peptide. It only requires an input SMILES and it automatically depicts the peptide structure with the labeled monomers. Optionally, the Norine graph annotation can be introduced in order to obtain the graph correctness. The generated image can be downloaded in svg or png formats. Apart from the visual results, the serialized monomer graph is also provided as a json file. The discovery mode is still not available in the current web service version.

## Conclusions

The usage of rBAN for Norine curation ended with the validation of 97% of the entries and the introduction of 242 SMILES and 11 monomers in the database. These results prove the ability of the algorithm to deduce effectively the monomeric graph of an NRP from its SMILES. The comparison with s2m and GRAPE clearly favored rBAN, which annotates more entries and with a higher perfomance. We also demonstrated the efficacy of the monomer discovery mode for the correction/addition of monomers. Furthermore, rBAN automatically produces graphs where the edges are labeled with the bond types linking the monomers. The current monomeric graphs in Norine do not contain this information, which is useful for the development of automatic substructure search. In the end, rBAN was integrated in Norine as a complementary tool for the future curation of the database. rBAN is accessible as a web service in Norine (http://bioinfo.cristal.univ-lille.fr/rban) and ExPASy (https://web.expasy.org/rban). The jar is publicly available on bitbucket (https://bitbucket.org/sib-pig/rban/downloads/).

### Limitations and perspectives

The main limitation of the method is its dependence to the defined fragmentation rules. Hence, it fails mapping natural products following different patterns such as Polyketides (PKs). The introduction of new rules based on PK biosynthesis patterns would solve this issue and would extend the range of secondary metabolites covered. The software currently provides the results in a JSON format but returning the graphs in specific annotation formats such as HELM or SCSR is planned in order to improve the usability of the tool. Finally, the current slow performance of the discovery mode will be addressed by trying alternative programmatic access to PubChem data or by downloading a part of the PubChem database to our local server. In future versions of the software it would also be interesting to include a modification database and implement an optional mapping where the monomers and their modifications are annotated independently.

## Additional files

**Additional file 1.** The file contains further details of the rBAN implementation and additional information of the analysis performed in the paper.

**Additional file 2.** Monomeric graph of Vancomycin. Example of a monomeric graph provided by rBAN.
