# Supplementary material for: rBAN: retro-biosynthetic analysis of nonribosomal peptides
Source: J Cheminform. 2019 Feb 8;11:13. doi: 10.1186/s13321-019-0335-x (PMC6689883; doi:10.1186/s13321-019-0335-x)
Supplement: Supplementary file 1 — Additional file 1. The file contains further details of the rBAN implementation and additional information of the analysis performed in the paper. [file 13321_2019_335_MOESM1_ESM.pdf]

rBAN: retro-biosynthentic analysis of non ribosomal peptides

Supplementary tables and figures

Table 1. Precomputation modifications.

Modification	Example
N-Methylation	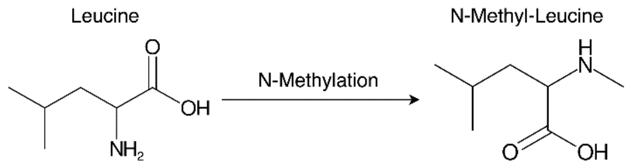
N-Formylation	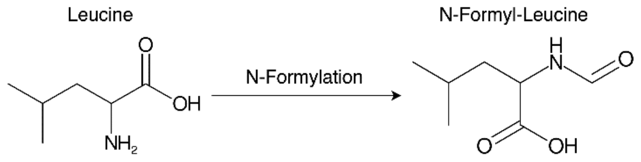
N-Acetylation	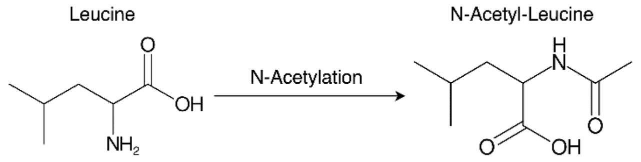
Amino group transfer	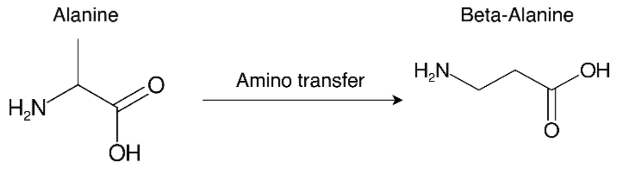

Table 2. Modifications of the fragments before the matching. The examples shown below represent the "in silico" process undertaken by rBAN to predict the original structure of the monomers. Note that they do not symbolise chemical equations.

<table>
<tr><th>Modification</th><th>Targets bonds</th><th>Example</th></tr>
<tr><td>Addition of hydroxyl group</td><td>Peptidic
Ester
Thioether
Carbon-Carboxyl
Carboxyl-Carboxyl
Glycosidic</td><td>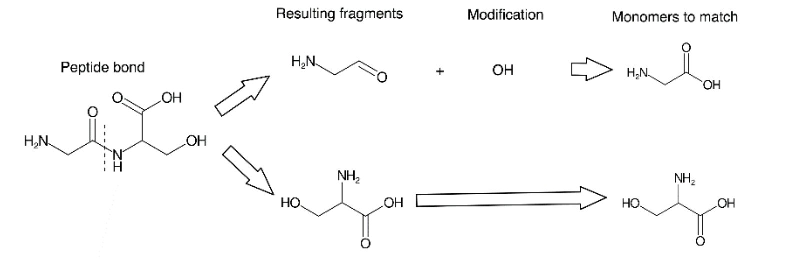
</td></tr>
<tr><td>Addition of oxygen</td><td>Peptide bonds with double bond character</td><td>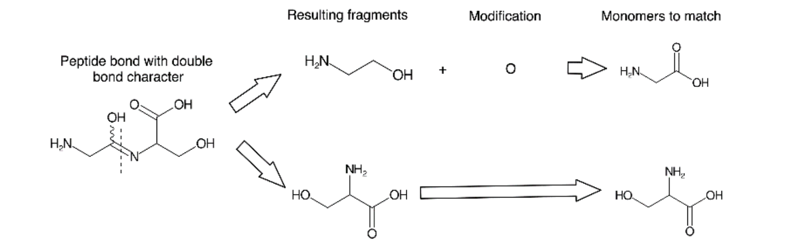
</td></tr>
<tr><td>Addition of oxygen and hydroxyl group</td><td>Heterocycle bonds</td><td>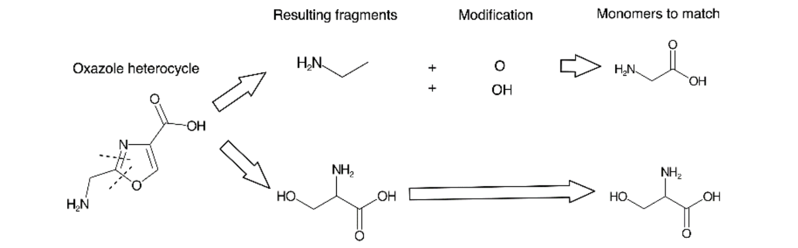
</td></tr>
</table>

Table 3. Suggested monomers NOT added in Norine (False positives).

Name	PubChemID	Structure	Compounds	Reason of false positive	References
**3-Buten-1-amine and 2-AMINOTHIAZOLE**	443732<br>2155	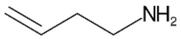<br>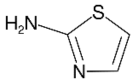	NOR00008, NOR00015,NOR00079, NOR00115 NOR00120, NOR00122, NOR00127, NOR00130 NOR00135, NOR00144, NOR00145, NOR00154 NOR00157, NOR00159, NOR00750, NOR00817	Wrong SMILES molecules	[1], [2],[3],[4],[5]
**2-Amino-4-methyl-pentanal**	4473091	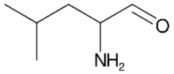	NOR00937, NOR00938, NOR00939, NOR00940	Wrong SMILES molecules	[6],[7],[8]
**dihydropyrrolecarbaldehyde**	18721951	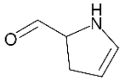	NOR00596,NOR00597	Wrong SMILES and wrong monomeric graph	[9]
**3-Methoxytyrosine**	1670	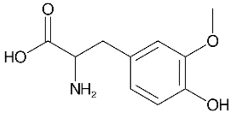	NOR00424, NOR00425	Wrong SMILES molecules	[10]
**Pentamide**	12298	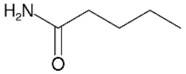	NOR00756, NOR01090	Wrong SMILES molecule (NOR00756) and wrong software annotation (NOR01090)	[11],[12]
**Isovaleric acid**	10430	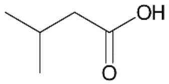	NOR00437	Wrong SMILES and wrong monomeric graph	[13]
**1,2-Dimethylpropylamine**	11731	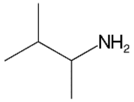	NOR00406, NOR00407	Wrong SMILES molecules	[14]

Fig. 1. Examples of Norine curation.

a) Correction of the monomeric graph of Destruxin A2 (NOR00068)

<u>Correction:</u>

Substitution of the monomer C4:1(3)-OH(2) for the new monomer suggested by rBAN: **C5:1(4)-OH(2)**

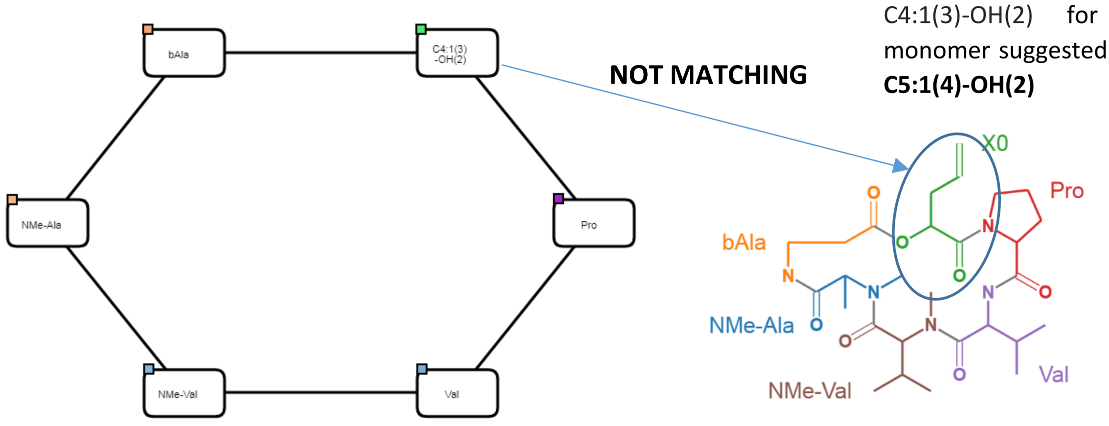

b) Correction of the SMILES of Guinamide D (NOR00437)

BEFORE **AFTER**

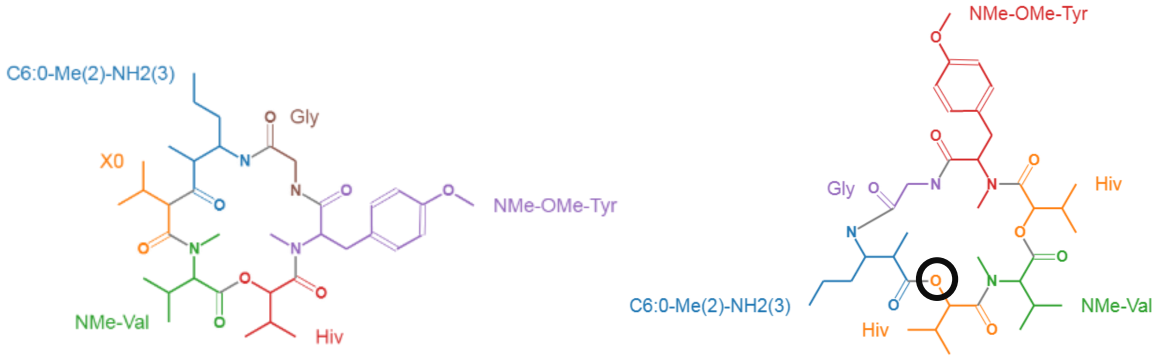

Fig. 2. Smiles2Monomers precomputation problem.

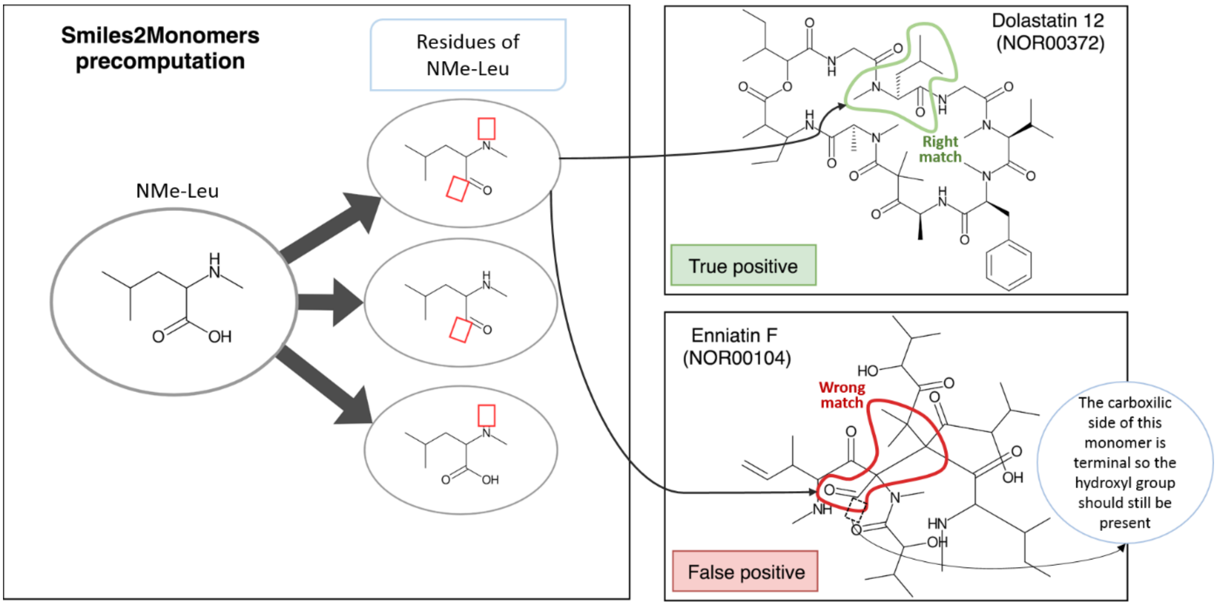

1. Psurek A, Neusüss C, Pelzing M, Scriba GK (2005) Analysis of the lipophilic peptaibol alamethicin by nonaqueous capillary electrophoresis-electrospray ionization-mass spectrometry. Electrophoresis 26:4368–4378

2. Zaharia IL, Gai Y, Zhou Y, Ward DE (2001) In planta sequential hydroxylation and glycosylation of a fungal phytotoxin: avoiding cell death and overcoming the fungal invader. Proc Natl Acad Sci 98:747–752

3. Namikoshi M, Yuan M, Sivonen K, et al (1998) Seven new microcystins possessing two L-glutamic acid units, isolated from Anabaena sp. strain 186. Chem Res Toxicol 11:143–149

4. Mitchell RE, Young H (1985) N-coronafacoyl-L-isoleucine and N-coronafacoyl-L-alloisoleucine, potential biosynthetic intermediates of the phytotoxin coronatine. Phytochemistry 24:2716–2717

5. Beattie KA, Kaya K, Sano T, Codd GA (1998) Three dehydrobutyrine-containing microcystins from Nostoc. Phytochemistry 47:1289–1292

6. Berek I, Becker A, Schröder H, et al (2009) Ampullosporin A, a peptaibol from Sepedonium ampullosporum HKI-0053 with neuroleptic-like activity. Behav Brain Res 203:232–239

7. Kronen M, Görls H, Nguyen H-H, et al (2003) Crystal structure and conformational analysis of ampullosporin A. J Pept Sci Off Publ Eur Pept Soc 9:729–744

8. Kronen M, Kleinwaechter P, Schlegel B, et al (2001) Ampullosporins B, C, D, E1, E2, E3 and E4 from Sepedonium ampullosporum HKI-0053: structures and biological activities. J Antibiot (Tokyo) 54:175–178

9. Gunasekera SP, Pomponi SA, McCarthy PJ (1994) Discobahamins A and B, new peptides from the Bahamian deep water marine sponge Discodermia sp. J Nat Prod 57:79–83

10. Ford PW, Gustafson KR, McKee TC, et al (1999) Papuamides A- D, HIV-Inhibitory and Cytotoxic Depsipeptides from the Sponges Theonella mirabilis and Theonella swinhoei Collected in Papua New Guinea. J Am Chem Soc 121:5899–5909

11. Laird DW, LaBarbera DV, Feng X, et al (2007) Halogenated cyclic peptides isolated from the sponge Corticium sp. J Nat Prod 70:741–746

12. Rinehart Jr KL, Gaudioso LA, Moore ML, et al (1981) Structures of eleven zervamicin and two emerimicin peptide antibiotics studied by fast atom bombardment mass spectrometry. J Am Chem Soc 103:6517–6520

13. Tan LT, Sitachitta N, Gerwick WH (2003) The Guineamides, Novel Cyclic Depsipeptides from a Papua New Guinea Collection of the Marine Cyanobacterium Lyngbya majuscula. J Nat Prod 66:764–771

14. Bringmann G, Lang G, Steffens S, Schaumann K (2004) Petrosifungins A and B, Novel Cyclodepsipeptides from a Sponge-Derived Strain of Penicillium brevicompactum. J Nat Prod 67:311–315
